# Rosacea and Its Association With Malignancy: Systematic Review

**DOI:** 10.2196/47821

**Published:** 2023-11-08

**Authors:** Luna Thapa, Joyce Xia, William Guo, Hunya Usmani, Devin Miller, Daniel Lozeau

**Affiliations:** 1 West Virginia School of Osteopathic Medicine Lewisburg, WV United States; 2 Renaissance School of Medicine at Stony Brook University Stony Brook, NY United States; 3 Department of Dermatology Renaissance School of Medicine at Stony Brook University Stony Brook, NY United States; 4 Stony Brook Dermatology Associates Stony Brook, NY United States

**Keywords:** rosacea, malignancies, skin cancer, glioma, breast cancer, hepatic cancer, thyroid cancer, systematic review

## Abstract

**Background:**

Rosacea is a chronic inflammatory skin condition that predominantly manifests as facial flushing, irritation, and acne. Rosacea and cancer are thought to be linked by the commonality of inflammatory and immune response dysfunction. Studies that have looked into this possible association have reported mixed results.

**Objective:**

Given the conflicting literature on this topic, our study sought to evaluate the overall association between rosacea and several cancers commonly investigated in the literature.

**Methods:**

A systematic review was conducted using the Cochrane, PubMed, Embase, and Ovid databases. Studies were screened independently for inclusion of rosacea and glioma and breast, thyroid, hepatic, or skin cancers. Using information from the articles, rosacea and each cancer were categorized as having a positive, negative, or unclear association.

**Results:**

Our systematic review included 39 full-text studies that investigated the association between rosacea and various malignancies. Among the malignancies of concern, 41% (16/39) of the studies reported an association with basal cell carcinoma, with 2 cohorts revealing an adjusted risk ratio (RR) of 1.50 (95% CI 1.35-1.67) and 0.72 (95% CI 0.56-0.93). In total, 33% (13/39) of the studies reported an association with squamous cell carcinoma, with 2 cohorts revealing an adjusted RR of 1.4 (95% CI 1.02-1.93) and 1.30 (95% CI 0.90-1.88). A total of 8% (3/39) of the studies reported an association between breast cancer and melanoma, with breast cancer cohorts revealing an adjusted RR of 8.453 (95% CI 1.638-43.606), 1.03 (95% CI 0.89-1.20), and 1.36 (95% CI 1.18-1.58) and melanoma cohorts revealing an adjusted RR of 1.10 (95% CI 0.95-1.27), 0.63 (95% CI 0.47-0.85), and 0.96 (95% CI 0.57-1.62). A total of 5% (2/39) of the studies reported an association among nonmelanoma skin cancers, hepatic cancer, and thyroid carcinomas, with nonmelanoma skin cancer cohorts revealing an adjusted RR of 1.36 (95% CI 1.26-1.47) and 2.66 (95% CI 1.53-4.61), hepatic cancer cohorts revealing an adjusted RR of 1.42 (95% CI 1.06-1.90) and 1.32 (95% CI 0.89-1.95), and thyroid carcinoma cohorts revealing an adjusted RR of 1.06 (95% CI 0.68-1.65) and 1.59 (95% CI 1.07-2.36). Only 1 cohort reported an association with glioma, revealing an adjusted RR of 1.36 (95% CI 1.18-1.58). According to our review, patients with rosacea were statistically more likely to have nonmelanoma skin cancers, breast cancer, and glioma. Rosacea was not found to be substantially associated with melanoma. The associations between rosacea and hepatic and thyroid cancers were unclear because of conflicting results.

**Conclusions:**

The current literature shows that rosacea is significantly associated with increased odds of nonmelanoma skin cancers, glioma, and breast cancer. Rosacea does not appear to be associated with melanoma. Further studies should be conducted to clarify the association between thyroid and hepatic cancers and rosacea.

## Introduction

### Background

Rosacea is a chronic inflammatory skin condition that predominantly manifests as persistent facial flushing, irritation, and acneiform papules or pustules. Although the etiology of rosacea remains unclear, a variety of factors both genetic and environmental seem to play a role in its pathogenesis. It has been hypothesized that elements of immune system dysregulation, deregulation of neurovascular signaling, and overgrowth of cutaneous pathogens are involved [1]. These abnormal inflammatory processes impair the skin’s ability to act as a protective barrier for the body [2]. A genetic component has been suggested as well given that rosacea tends to appear more frequently in patients of Northern European and Celtic ancestry [1]. As the pathogenesis of rosacea is known to involve an abnormal inflammatory response, studies have been conducted to investigate its co-occurrence with other systemic diseases. Recent studies have suggested that rosacea is associated with a heightened risk of various chronic systemic diseases, including hypertension, autoimmune disease, cardiovascular disease, gastrointestinal disorders, and dyslipidemia [3], as well as multiple psychiatric comorbidities (major depressive disorder, persistent mood disorders, adjustment disorder, and generalized anxiety disorder) [4]. The complex relationship between inflammation and mental health is yet to be clearly understood, but there is evidence that chronic low-grade inflammation can contribute to mental illnesses such as depression [5]. Therefore, the inflammatory qualities of a chronic disease such as rosacea can potentially have far-reaching effects on patients’ mental and physical health.

In addition to the aforementioned illnesses, there have also been many studies that have examined the association between rosacea and various cancers. The current understanding of the pathophysiology of this relationship is limited. However, it is hypothesized that patients with rosacea may have an increased risk of skin cancer because of inflammatory changes in the skin barrier, including reduced epidermal levels of photoreceptive transurocanic acid [6].

### Objectives

Studies that have investigated the relationship between rosacea and cancer have reported inconsistent results. A recent study in Denmark [3,6] found that patients with rosacea had an increased risk of developing glioma, nonmelanoma skin cancer (NMSC), breast cancer, and hepatic cancer, whereas another study in the United States noted that females who had a history of rosacea had a subsequently increased risk of thyroid and basal cell carcinoma (BCC) [7]. Dupont [8], in contrast, found no substantial association between rosacea and skin cancers. Given the ambiguity of these findings, we conducted a systematic review of the current published work to evaluate the relationship between rosacea and various commonly studied cancers in the literature, including BCC; squamous cell carcinoma (SCC); Merkel cell carcinoma; melanoma; glioma; and hepatic, breast, and thyroid carcinomas.

## Methods

### Eligibility and Criteria

In accordance with the PRISMA (Preferred Reporting Items for Systematic Reviews and Meta-Analyses) guidelines, we conducted a review of observational studies on the association between rosacea and various cancers. Case reports, case series, case-control studies, cross-sectional studies, and cohort studies were included.

### Literature Search and Study Selection

The Cochrane, PubMed, Embase, and Ovid databases were searched for relevant studies from inception to March 3, 2021. The search was then updated on June 9, 2023. The search terms are provided in detail in Multimedia Appendix 1.

Studies were included or excluded from a meta-analysis based on the criteria outlined in Textbox 1.

All studies were screened by 2 independent reviewers (LT and JX), and eligibility for inclusion was determined by screening the titles and reviewing the full texts. Any conflicts of eligibility were resolved by a third-party reviewer (WG). Using information obtained from the articles, rosacea and each cancer were categorized as likely or unlikely associated, whereas cancers with conflicting results were categorized as having an unclear association.

**Inclusion and exclusion criteria.**

**Inclusion criteria**

1. Studies that investigated patients with a diagnosis of basal cell carcinoma; nonmelanoma skin cancer; squamous cell carcinoma; Merkel cell carcinoma; melanoma; glioma; or hepatic, breast, or thyroid carcinoma and a diagnosis of rosacea
2. Patients diagnosed with erythematotelangiectatic rosacea, papulopustular (or acne) rosacea, rhinophyma, or ocular rosacea
3. Papers studying populations in different countries
4. Case reports; series; and case-control, cohort, or cross-sectional studies
5. Patients of all ages, sexes, and nationalities

**Exclusion criteria**

1. Papers for which full text was not available
2. N<1 patients
3. Papers not written in English
4. Studies conducted on nonhuman subjects
5. Studies including concurrent acne and rosacea
6. Studies including diagnoses of perioral dermatitis

### Data Extraction and Risk-of-Bias Assessment

Data extracted from the included studies comprised author, publication year, title, study location, study type, total number of patients, number of patients with rosacea vs control patients without rosacea, general mean age, mean age of patients presenting with rosacea, general percentage of female patients, percentage of female patients presenting with rosacea, number of patients who presented with a history of smoking, drinking, confounding diseases, type of rosacea, inclusion and exclusion criteria used for the study, method of diagnosis of rosacea, mean duration of rosacea, socioeconomic status association with prevalent rosacea, number of patients with cancer in general, number of patients with cancer and rosacea, *P* value, odds ratio (OR; 95% CI), hazard ratio (HR), incidence rate ratio, risk ratio (RR), and any adjustments made to the statistical values. To assess the risk of bias of the included studies, the Newcastle-Ottawa Quality Assessment Scale was used.

## Results

### Search Results

**Figure 1:**
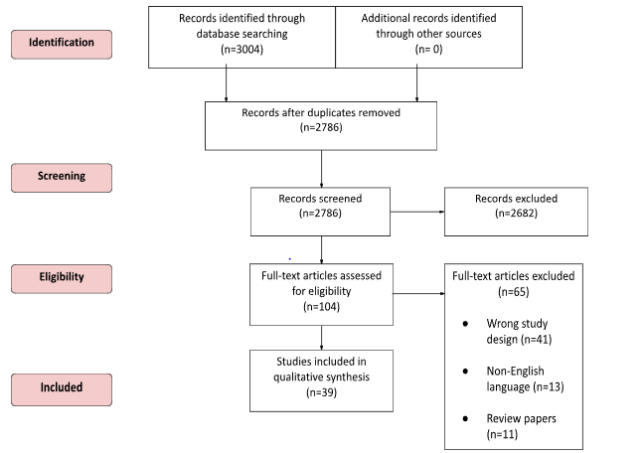
PRISMA (Preferred Reporting Items for Systematic Reviews and Meta-Analyses) diagram for the review on rosacea and its association with various cancers.

Among the 3004 articles originally identified, 2786 (92.74%) remained after deduplication and were screened. After initial screening, of the 2786 remaining articles, 104 (3.73%) full-text articles were assessed for eligibility, of which 39 (37.5%) were eligible for our systematic review (Table 1). A total of 62.5% (65/104) of the full texts were excluded for reasons such as being in a non-English language, being the wrong publication type (including conference abstracts), having the wrong study design (including research papers), or looking at the wrong outcomes (Figure 1). Owing to a lack of available studies, we were not able to conduct a meta-analysis.

**Table 1:** Characteristics of the studies.

Cancer type	Study	Title (study type)	Total number of patients	Age (years), mean (SD)	Female patients	Method of cancer diagnosis	Inclusion and exclusion criteria	Statistical results
BCC^a^	Li et al [7], 2015	Personal history of rosacea and risk of incident cancer among women in the United States (prospective-retrospective mixed cohort study)	75,088 (United States)	Rosacea: 37.6 (4.1); control: 36.2 (4.7)	100%	Pathologically confirmed invasive cases via medical record and self-report	NHS II^b^ records excluding records for which there was missing date of birth, record of all cancers at baseline, and all responses from racial and ethnic minority people	Outcome: BCC was found to be significantly higher among patients with rosacea as compared with controls; adjusted RR^c^=1.50 (95% CI 1.35-1.67) and *P*<.05; adjusted for age, BMI, alcohol consumption, physical activity, physical examination, multivitamin use, smoking status, oral contraceptive use, menopausal status, postmenopausal hormone use, and use of medications (including tetracycline, isotretinoin, and antibiotics)
BCC	Lin et al [9], 2023	Single-institution retrospective study evaluating personal history of rosacea and risk of BCC of the face (retrospective, population-based cohort study)	4537 (United States)	Nonrosacea: 72.4 (12.8); rosacea: 72.2 (12.3)	Nonrosacea: 1048 (45%); rosacea: 59 (47.6%)	Confirmed via the ICD-10^d^ code, whereas 267 had a history of provider- or patient-reported rosacea according to available medical records	N/A^e^	Outcome: BCC occurring on the face or head and history of rosacea were significantly lower than in patients without history of rosacea; aOR^f^ 0.72 (95% CI 0.56-0.93); facial BCC: *P*<.001; nonfacial BCC: *P*<.58; adjusted for multivariate logistic regression analysis adjusted for age, sex, smoking history, and skin of color
SCC^g^	Lin et al [10], 2022	Prospective study evaluating the personal history of rosacea and risk of cutaneous SCC among women in the United States (retrospective, population-based cohort study)	90,238 (United States)	Nonrosacea: 36.1 (4.7); rosacea: 37.6 (4.1)	N/A	Study participants completed biennial questionnaires that gathered medical history, including clinician-diagnosed cSCC^h^ and rosacea. During the follow-up, 577 cSCC cases were documented and confirmed via pathology reports.	N/A	Outcome: rosacea had an overall significant association with cSCC, especially when developed on the head and neck. Rosacea had no significant association with non–head and neck SCC; adjusted RR=1.4 (95% CI 1.02-1.93); Cox proportional hazard model and multivariate model with adjustments for age and other cancer risk factors
SCC	Li et al [7], 2015	Personal history of rosacea and risk of incident cancer among women in the United States (prospective-retrospective mixed cohort study)	75,088 (United States)	Rosacea: 37.6 (4.1); control: 36.2 (4.7)	100%	Pathologically confirmed invasive cases via medical record and self-report	NHS II records excluding records for which there was missing date of birth, record of all cancers at baseline, and all responses from racial and ethnic minority people	Outcome: there were no statistically significant associations found between rosacea and SCC; adjusted HR^i^=1.30 (95% CI 0.90-1.88); adjusted for age, BMI, alcohol consumption, physical activity, physical examination, multivitamin use, smoking status, oral contraceptive use, menopausal status, postmenopausal hormone use, and use of medications (including tetracycline, isotretinoin, and antibiotics)
NMSC^j^	Egeberg et al [6], 2017	Rosacea and risk of cancer in Denmark (retrospective, population-based cohort study)	4,361,688 (Denmark)	Rosacea: 53.7 (16.5); control: 48.6 (18.0)	Rosacea: 68%; control: 50.6%	ICD-10 code C73 in DNPR^k^ medical record	All Danish adults (aged 18 years) alive and residing in Denmark on January 1, 2008. Patients were followed up from study start until December 31, 2012; death; migration; or the occurrence of an end point, whichever came first.	Outcome: patients diagnosed with rosacea were statistically more likely to develop NMSC compared with patients in the reference population; adjusted HR=1.36 (95% CI 1.26-1.47) and *P*<.001; adjusted for age, sex, SES^l^, and health care consumption
NMSC	Cho et al [11], 2022	Risk of Skin Cancer and Actinic Keratosis in Patients with Rosacea: A Nationwide Population-based Cohort Study (retrospective, population-based cohort study)	11,420 (South Korea)	61.2% (n=6698) were in the age range of 40 to 59 years.	64.7% (n=7092)	NHISS^m^ in South Korea was used to compile claims from January 2010 to December 2019 with ≥1 relevant ICD-10 codes (actinic keratosis: L570; KC^n^: C44 and D04; melanoma: C43 and D03; gastric cancer: C16; colorectal cancer: C18, C19, and C20; and liver cancer: C22)	Patients with preexisting principal diagnoses of the target disease before the index date were excluded. To establish a well-matched control group, they used specific criteria, including sex, age, income, and residence, at a 1:2 ratio. Any study patients lacking suitable matched controls and control patients with previous target disease diagnoses were subsequently excluded from the analysis. The index date for patients with rosacea was set as the date of their initial diagnosis, with control patient index dates adjusted accordingly. The observation period extended until December 31, 2019, and individuals who passed away without experiencing the target disease diagnosis were censored.	Outcome: rosacea had a significant association with the development of NMSC; adjusted HR=2.66 (95% CI 1.53-4.61); multivariable stratified Cox proportional hazard model
Breast cancer	Egeberg et al [6], 2017	Rosacea and Risk of Cancer in Denmark (retrospective, population-based cohort study)	4,361,688 (Denmark)	Rosacea: 53.7 (16.5); control: 48.6 (18.0)	Rosacea: 68%; control: 50.6%	ICD-10 code C73 in DNPR medical record	All Danish adults (aged 18 years) alive and residing in Denmark on January 1, 2008. Patients were followed up from study start until December 31, 2012; death; migration; or the occurrence of an end point, whichever came first.	Outcome: patients with rosacea were more likely to develop breast cancer compared with patients without a history of rosacea; aOR 8.453 (95% CI 1.638-43.606) and *P*=.01; adjusted for multivariate model
Breast cancer	Li et al [12], 2020	Epidemiological features of rosacea in Changsha, China: A population‐ based, cross‐sectional study (population-based cross-sectional case-control study)	794 analyzed and 10,095 enrolled (China)	Overall: 35.5 (19.1); female patients with rosacea: 37.4 (10.1); male patients with rosacea: 42.8 (15.2); patients with rosacea overall: 38.4 (11.3)	Rosacea: 82.61%; control: 70.65%	Previous diagnosis at clinics or superior-level hospitals	5 randomly selected neighborhoods that were then classified into socioeconomic strata (rich, moderate, and poor), with one community from each stratum selected. All Han Chinese citizens from these 15 communities with 4025 families and 12,775 individuals were included. Of these, 628 families chose not to attend the interview and were excluded from the study. The final sample size was 10,095.	Outcome: no significant increase in the occurrence of breast cancer in patients with rosacea; adjusted RR=1.03 (95% CI 0.89-1.20); adjusted for age, BMI, alcohol consumption, physical activity, physical examination, multivitamin use, smoking status, oral contraceptive use, menopausal status, postmenopausal hormone use, history of severe teenage acne, and use of medications (including tetracycline, isotretinoin, and antibiotics). Additionally adjusted for personal history of benign breast disease, family history of breast cancer, age at first birth and parity, age at menarche, height, and BMI at the age of 18 years.
Breast cancer	Li et al [7], 2015	Personal history of rosacea and risk of incident cancer among women in the United States (prospective-retrospective mixed cohort study)	75,088 (United States)	Rosacea: 37.6 (4.1); control: 36.2 (4.7)	100%	Pathologically confirmed invasive cases via medical record and self-report	NHS II records excluding records for which there was missing date of birth, record of all cancers at baseline, and all responses from racial and ethnic minority people	Outcome: female patients with a diagnosis of rosacea were more likely to have had breast cancer; however, male patients with rosacea had no significant increase in breast cancer incidence; IRR^o^ (person-years): overall=1.36 (95% CI 1.18-1.58); females=1.27 (95% CI 1.05-1.54); males=1.47 (95% CI 1.17-1.84); overall: *P*<.001; females: *P*=.02; males: *P*<.001; IRR adjusted for age, sex, and SES
Glioma	Egeberg et al [3], 2016	Association of Rosacea With Risk for Glioma in a Danish Nationwide Cohort Study (nationwide cohort study)	5,416,138 (Denmark)	Rosacea: 42.2 (16.5); control: 40.8 (19.7)	Rosacea: 67.3%; control: 50.4%; females: 45,994 (67.3%) with rosacea and 2,732,029 (50.4%) controls; males: 22,378 (32.7%) with rosacea and 2,684,509 (49.6%) controls	Hospital diagnosis of glioma (ICD-8^p^ code 191 and ICD-10 codes C71, D33, and D43) recorded in the DNPR	Inclusion: Danish citizens aged ≥18 years on January 1, 1997, or the subsequent day they reached 18 years. Individuals were followed up until December 31, 2011; migration; a diagnosis of glioma; or death owing to any cause, whichever came first. Exclusion criteria: patients with rosacea or glioma at baseline	Outcome: although the correlation was weak, the study found a significantly increased risk of glioma in patients with rosacea; rosacea-associated increased risk of glioma was greater in males than in females; IRR (person-years): overall=1.36 (95% CI 1.18-1.58); females=1.27 (95% CI 1.05-1.54); men=1.47 (95% CI 1.17-1.84); overall: *P*<.001; females: *P*=.02; males: *P*<.001; IRR adjusted for age, sex, and SES
Hepatic cancer	Egeberg et al [6], 2017	Rosacea and Risk of Cancer in Denmark (retrospective, population-based cohort study)	4,361,688 (Denmark)	Rosacea: 53.7 (16.5); control: 48.6 (18.0)	Rosacea: 68%; control: 50.6%	ICD-10 code C73 in DNPR medical record	All Danish adults (aged 18 years) alive and residing in Denmark on January 1, 2008. Patients were followed up from study start until December 31, 2012; death; migration; or the occurrence of an end point, whichever came first.	Outcome: patients with rosacea had an increased risk of developing hepatic cancer; adjusted HR=1.42 (95% CI 1.06-1.90) and *P*=.02; adjusted for age, sex, SES, and health care consumption
Hepatic cancer	Cho et al [11], 2022	Risk of Skin Cancer and Actinic Keratosis in Patients with Rosacea: A Nationwide Population-based Cohort Study (retrospective, population-based cohort study)	11,420 (South Korea)	61.2% (n=6698) were in the age range of 40 to 59 years.	64.7% (n=7092)	NHISS in South Korea was used to compile claims from January 2010 to December 2019 with ≥1 relevant ICD-10 codes (actinic keratosis: L570; KC: C44 and D04; melanoma: C43 and D03; gastric cancer: C16; colorectal cancer: C18, C19, and C20; and liver cancer: C22)	Patients with preexisting principal diagnoses of the target disease before the index date were excluded. To establish a well-matched control group, they used specific criteria, including sex, age, income, and residence, at a 1:2 ratio. Any study patients lacking suitable matched controls and control patients with previous target disease diagnoses were subsequently excluded from the analysis. The index date for patients with rosacea was set as the date of their initial diagnosis, with control patient index dates adjusted accordingly. The observation period extended until December 31, 2019, and individuals who passed away without experiencing the target disease diagnosis were censored.	Outcome: rosacea had no significant association with the development of hepatic cancer; adjusted HR=1.32 (95% CI 0.89-1.95); multivariable stratified Cox proportional hazard model
Thyroid cancer	Egeberg et al [6], 2017	Rosacea and Risk of Cancer in Denmark (retrospective, population-based cohort study)	4,361,688 (Denmark)	Rosacea: 53.7 (16.5); control: 48.6 (18.0)	Rosacea: 68%; control: 50.6%	ICD-10 code C73 in DNPR medical record	All Danish adults (aged 18 years) alive and residing in Denmark on January 1, 2008. Patients were followed up from study start until December 31, 2012; death; migration; or the occurrence of an end point, whichever came first.	Outcome: no significant increase in the development of thyroid cancer in patients with rosacea; adjusted HR=1.06 (95% CI 0.68-1.65) and *P*=.80; adjusted for age, sex, SES, and health care consumption
Thyroid cancer	Li et al [7], 2015	Personal history of rosacea and risk of incident cancer among women in the United States (prospective-retrospective mixed cohort study)	75,088 (United States)	Rosacea: 37.6 (4.1); control: 36.2 (4.7)	100%	Pathologically confirmed invasive cases via medical record and self-report	NHS II records excluding records for which there was missing date of birth, record of all cancers at baseline, and all responses from racial and ethnic minority people	Outcome: found an increase in the development of thyroid cancer in patients with rosacea; adjusted RR=1.59 (95% CI 1.07-2.36) and *P*<.05; adjusted for age, BMI, alcohol consumption, physical activity, physical examination, multivitamin use, smoking status, oral contraceptive use, menopausal status, postmenopausal hormone use, and use of medications (including tetracycline, isotretinoin, and antibiotics)
Melanoma	Egeberg et al [6], 2017	Rosacea and Risk of Cancer in Denmark (retrospective, population-based cohort study)	4,361,688 (Denmark)	Rosacea: 53.7 (16.5); control: 48.6 (18.0)	Rosacea: 68%; control: 50.6%	ICD-10 code C73 in DNPR medical record	All Danish adults (aged 18 years) alive and residing in Denmark on January 1, 2008. Patients were followed from study start until December 31, 2012; death; migration; or the occurrence of an end point, whichever came first.	Outcome: no increased occurrence of melanoma in patients with rosacea; adjusted HR=1.10 (95% CI 0.95-1.27) and *P*=.19; adjusted for age, sex, SES, and health care consumption
Melanoma	Erickson et al [13], 2019	Sex differences for incident cancer in patients with rosacea: Real-world evidence from a large Midwestern US patient population (retrospective, population-based cohort study)	186,829 (United States)	N/A	Overall: 76% diagnosed with cancer of some type (not necessarily melanoma)	N/A	A medical record data repository (>6 million patients) was searched (using ICD-9^q^ and ICD-10 codes) for data from patients of dermatology with a rosacea diagnosis vs patients without rosacea as a control population (≥1-year follow-up; January 2001-November 2018) and who had a subsequent diagnosis of any cancer	Outcome: cutaneous melanoma was inversely associated with rosacea in female patients (aOR 0.63, 95% CI 0.47-0.85; *P*=.003; IRR=10.41 per 10,000 females/y) but not in male patients; adjusted for age and race
Melanoma	Li et al [7], 2015	Personal history of rosacea and risk of incident cancer among women in the United States (prospective-retrospective mixed cohort study)	75,088 (United States)	Rosacea: 37.6 (4.1); control: 36.2 (4.7)	100%	Pathologically confirmed invasive cases via medical record and self-report	NHS II records excluding records for which there was missing date of birth, record of all cancers at baseline, and all responses from racial and ethnic minority people	Outcome: no significant increase in the development of malignant melanoma in patients with rosacea; adjusted RR=0.96 (95% CI 0.57-1.62); adjusted for age, BMI, alcohol consumption, physical activity, physical examination, multivitamin use, smoking status, oral contraceptive use, menopausal status, postmenopausal hormone use, and use of medications (including tetracycline, isotretinoin, and antibiotics)
Melanoma	Cho et al [11], 2022	Risk of Skin Cancer and Actinic Keratosis in Patients with Rosacea: A Nationwide Population-based Cohort Study (retrospective, population-based cohort study)	11,420 (South Korea)	61.2% (n=6698) were in the age range of 40 to 59 years.	64.7% (n=7092)	NHISS in South Korea was used to compile claims from January 2010 to December 2019 with ≥1 relevant ICD-10 codes (actinic keratosis: L570; KC: C44 and D04; melanoma: C43 and D03; gastric cancer: C16; colorectal cancer: C18, C19, and C20; and liver cancer: C22)	Patients with preexisting principal diagnoses of the target disease before the index date were excluded. To establish a well-matched control group, they used specific criteria, including sex, age, income, and residence, at a 1:2 ratio. Any study patients lacking suitable matched controls and control patients with previous target disease diagnoses were subsequently excluded from the analysis. The index date for patients with rosacea was set as the date of their initial diagnosis, with control patient index dates adjusted accordingly. The observation period extended until December 31, 2019, and individuals who passed away without experiencing the target disease diagnosis were censored.	Outcome: rosacea had no significant association with the development of melanoma; adjusted HR=1.69 (95% CI 0.25-11.37); multivariable stratified Cox proportional hazard model

^a^BCC: basal cell carcinoma.

^b^NHS II: Nurses’ Health Study II.

^c^RR: risk ratio.

^d^ICD-10: International Classification of Diseases, 10th Revision.

^e^N/A: not applicable.

^f^aOR: adjusted odds ratio.

^g^SCC: squamous cell carcinoma.

^h^cSCC: cutaneous squamous cell carcinoma.

^i^HR: hazard ratio.

^j^NMSC: nonmelanoma skin cancer.

^k^DNPR: Danish National Patient Register.

^l^SES: socioeconomic status.

^m^NHISS: National Health Insurance Sharing Service.

^n^KC: keratinocyte carcinoma.

^o^IRR: incidence rate ratio.

^p^ICD-8: International Classification of Diseases, Eighth Revision.

^q^ICD-9: International Classification of Diseases, Ninth Revision.

### Risk of Bias of the Included Studies

**Figure 2:**
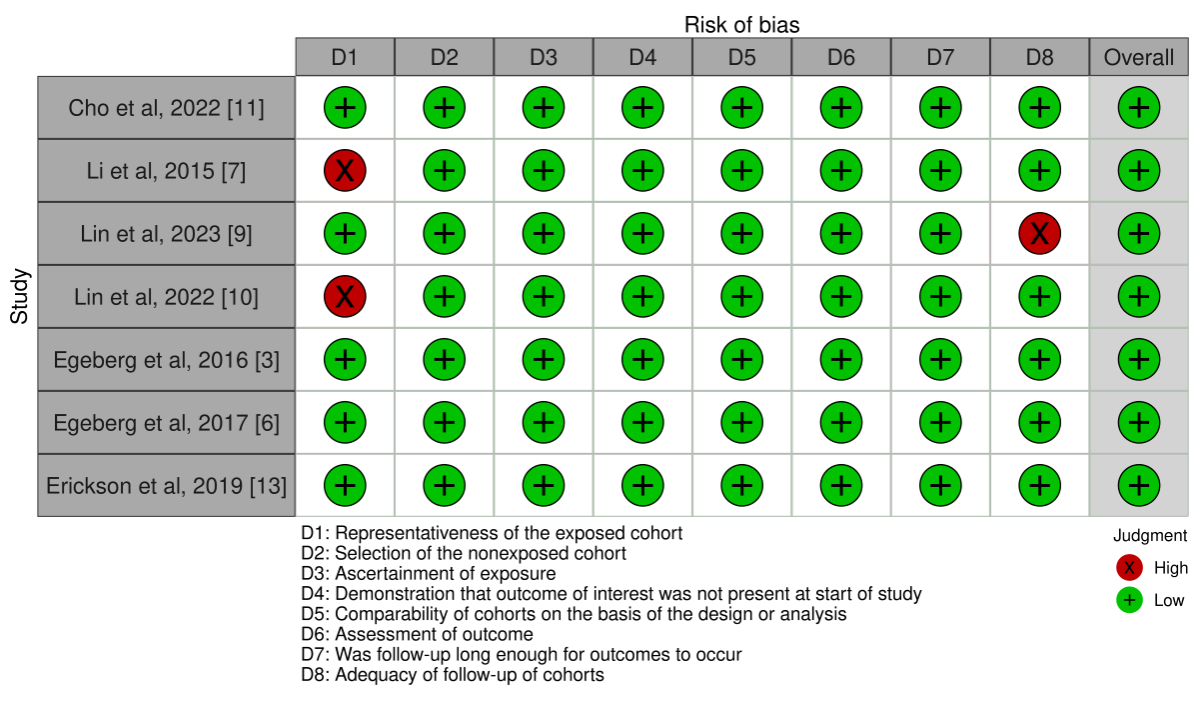
Detailed Newcastle-Ottawa Quality Assessment Scale of each included cohort study [3,6,7,9,10,11,13,14].

**Figure 3:**
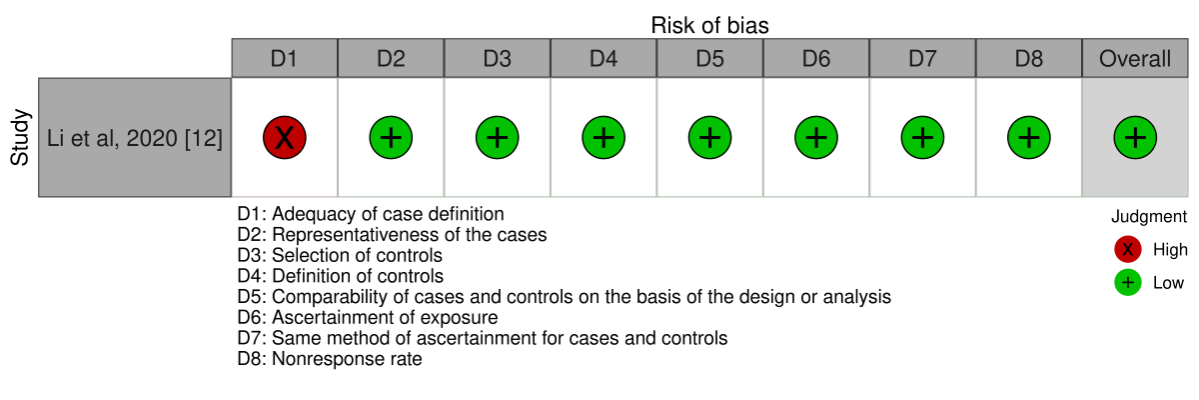
Detailed Newcastle-Ottawa Quality Assessment Scale of each included case-control study [12,14].

The risk of bias of the studies included for the statistical analysis is summarized in Figures 2 [3,6,7,9-11,13] and 3 [12]. The Newcastle-Ottawa Quality Assessment Scale was used to assess the bias found in a total of 21% (8/39) of the studies included for analysis. Of these 8 studies, 7 (88%) were rated as having a low risk of bias based on a Newcastle-Ottawa Quality Assessment Scale for cohort studies score of ≥5 (Figure 2). There was 1 study for which we had to use the Newcastle-Ottawa Quality Assessment Scale for case-control studies and that was also determined to have a low risk of bias with a score of ≥5 (Figure 3).

### Rosacea and BCC

Our search returned 41% (16/39) of papers examining the relationship between rosacea and BCC. Of these 16 papers, 2 (12%) were cohort studies, the first investigating the impact of a personal history of rosacea on the risk of developing BCC in females in the United States and the second investigating the impact of a personal history of rosacea on the risk of developing facial BCC in females in the Providence, Rhode Island, region [7]. In the first study, 424 patients with rosacea and 4552 age- and sex-matched controls without rosacea were followed up for reports of the development of cancer. The mean age of the study sample was 37.6 (SD 4.1) years for patients with rosacea and 36.2 (4.7) years for the control group. The prevalence of BCC was found to be significantly higher among patients with rosacea compared with controls [7].

In the second study, of the 4537 patients diagnosed with BCC from October 2016 to November 2020 in the Rhode Island region, 2453 had BCC on the face, and of them, 267 had a history of rosacea. A multivariate model with adjustments for age, sex, smoking history, skin color, and other cancer risk factors was developed. Results for chi-square and *R* statistic analysis indicated that facial BCC in patients with a history of rosacea was significantly lower than in patients without rosacea (3.80 vs 5.07 per 100 patients; *P*<.001). When comparing BCC of the body, no significant difference was found between patients with a history of rosacea and patients without rosacea (3.95 vs 4.22 per 100 patients; *P*<.58) [9].

The other 88% (14/16) of papers on rosacea and BCC comprised case series or case reports. Notably, all reported cases detailed male patients with rhinophymatous rosacea who were later diagnosed with BCC in the area of the nodular lesion (Table 2). Lazzeri et al [15] presented a case series and literature review in which they found a total of 46 patients (including 3 new cases of their own) with rhinophyma that went on to develop cutaneous cancer. Of these patients, 28 were diagnosed with BCC, 11 were diagnosed with SCC, 4 were diagnosed with SCC and BCC, and 1 was found to have angiosarcoma. Common presenting symptoms in these patients included sudden progressive enlargement of long-standing rhinophyma in both BCC and SCC cases and additional symptoms of ulceration, malodorous drainage, and serous discharge in SCC cases [15]. Interestingly, BCC was less likely than SCC to present with sudden ulceration, bleeding, or serous discharge, although such symptoms have certainly been observed [16-20].

**Table 2:** Characteristics of case series and reports that investigated basal cell carcinoma (BCC) and squamous cell carcinoma (SCC) in patients with rosacea.

Study, year	Cancer type	Cases, n	Type of preexisting rosacea	Geographic location
Acker and Helwig [21], 1967	BCC	5	Rhinophyma	Washington, DC
Baruchin et al [22], 1998	BCC	4	Rhinophyma	Israel
Keefe et al [17], 1988	BCC	1	Rhinophyma	Scotland
Kwah and Lawrence [18], 2011	BCC	1	Rhinophyma	North Carolina
Lazzeri et al [15], 2012	BCC	2	Rhinophyma	Pisa, Italy
Leyngold et al [23], 2008	BCC	1	Rhinophyma	Nevada, United States
McKenna and McKenna [19], 2006	BCC	1	Rhinophyma	Northern Ireland, United Kingdom
Nambi et al [20], 2008	BCC	1	Rhinophyma	Saudi Arabia
Plenk [24], 1995	BCC	2	Rhinophyma	Utah, United States
Rees [25], 1955	BCC	2	Rhinophyma	New York, United States
Silvis and Zachary [26], 1990	BCC	1	Rhinophyma	Minneapolis, United States
Zabbia et al [27], 2014	BCC	6	Rhinophyma	Palmero, Italy
Kornblut and Evers [28], 1973	SCC	1	Rhinophyma	Maryland, United States
Broadbent and Cort [29], 1977	SCC	2	Rhinophyma	England
Barankin [16], 2005	BCC	1	Rhinophyma	Canada
Jain et al [30], 1973	SCC	1	Rhinophyma	Udaipur, India
Kesty and Baldwin [31], 2017	SCC	1	Rhinophyma	Florida, United States
Lazzeri et al [15], 2012	SCC	1	Rhinophyma	Pisa, Italy
Lutz and Otley [32], 2001	SCC	1	Rhinophyma	Florida, United States
Rizzi et al [33], 2016	SCC	1	Rosacea	Milan, Italy
Ross and Davies [34], 1991	SCC	1	Rhinophyma	London, United Kingdom
Tamir et al [35], 1999	SCC and BCC	1	Rhinophyma	Washington, United States
Theotoka et al [36], 2020	SCC	2	Ocular rosacea	Miami, United States
Chlebicka et al [37], 2021	BCC	2	Rhinophyma	Wrocław, Poland

### Rosacea and SCC

Our search returned 33% (13/39) of studies examining the relationship between rosacea and SCC. Of these, one was a cohort study investigating the impact of a personal history of rosacea on the risk of developing SCC in females in the United States. In this study, 577 cases of SCC were isolated from a population of 90,238 females. A Cox proportional hazard model and multivariate model with adjustments for age and other cancer risk factors were developed. The results indicated that rosacea was significantly associated with overall cutaneous SCC (RR 1.40, 95% CI 1.02-1.93). In this study, the location of SCC was also divided into 2 groups—head and neck and non–head and neck—which were also compared with the history of rosacea. The results indicated that rosacea was significantly associated with head and neck SCC (RR 1.71, 95% CI 1.09-2.69). Findings indicated no significant statistical associations between rosacea and non–head and neck SCC (RR 1.21, 95% CI 0.78-1.90) [10].

The second study investigated various cancer risks among females in the United States with a history of rosacea. Among a total of 75,088 females, 6015 patients were diagnosed with rosacea from 1991 to 2011, with 452 SCC diagnoses documented. A Cox proportional hazard model and multivariate model with adjustments for age, BMI, alcohol consumption, smoking history, and other cancer risk factors were developed. The results indicated that there were no statistically significant associations between rosacea and SCC (HR 1.30, 95% CI 0.90-1.88) [7].

The other 85% (11/13) of the studies were case series and reports that examined the association between rosacea and SCC. Again, all studies identified SCC development in patients with long-standing rhinophyma (Table 2). Notably, Kesty and Baldwin [31] reported the case of a patient aged 67 years with no previous history of cancer who presented with an enlarging left nasal mass of 4 months. An initial punch biopsy diagnosed rhinophyma, and the patient was sent home. The patient presented back to the hospital several weeks later with new reports of ulceration, drainage, and rapid growth of the mass and had another biopsy indicating invasive SCC. Although a total rhinectomy could be performed, the patient was ultimately found to have metastases to the lungs and submandibular area [31]. Although such cases are rare and no overall association can be made, these reports may serve as a warning to physicians to remain vigilant for the possibility of carcinoma in patients with rhinophyma, particularly those with rapidly growing and ulcerating masses.

### Rosacea and NMSC

Our search returned 5% (2/39) of studies that did not differentiate between NMSCs. In the first case, Egeberg et al [6] conducted a cohort study analyzing data from the nationwide Danish registry. The study included 49,475 patients with rosacea with an average age of 53.7 (SD 16.5) years and a reference population of 4,312,213 patients without rosacea with an average age of 48.6 (SD 18.0) years. Their findings noted that patients diagnosed with rosacea were statistically more likely to develop NMSC compared with patients in the reference population (HR 1.36, 95% CI 1.26-1.47) [6]. In the second case, Cho et al [11] conducted a nationwide population-based retrospective cohort study in South Korea. The study included 11,420 patients compared using a multivariable stratified Cox proportional hazard model. Their findings noted that patients with rosacea had a significant association with the development of NMSC compared with the reference population (HR 2.66, 95% CI 1.53-4.61) [11].

### Rosacea and Breast Cancer

A total of 8% (3/39) of the studies analyzed the association between rosacea and breast cancer. Of these 3 studies, 2 (67%) were cohort studies. Egeberg et al [6] conducted a cohort study that analyzed data from the nationwide Danish registry and found that patients with rosacea were more likely to develop breast cancer compared with patients without a history of rosacea (HR 1.25, 95% CI 1.15-1.36). However, Li et al [7] found no significant increase in the occurrence of breast cancer in patients with rosacea (RR 1.03, 95% CI 0.89-1.20).

Li et al [12] also conducted a case-control study to determine the present epidemiological status of rosacea in China. From a total of 10,095 patients enrolled in the study, 351 were observed to have rosacea, 290 of whom were female and 61 of whom were male. The overall average age of patients with rosacea was 38.4 (SD 11.3) years, whereas the average age of the control population was 35.5 (SD 19.1) years. A previous diagnosis of melasma, hypertension, coronary heart disease, hyperthyroidism, diabetes, chronic gastritis, peptic ulcer, gastrointestinal cancer, breast cancer, and gynecological cancer was then identified in patients in both the control and rosacea populations. It was found that female patients with a diagnosis of rosacea were more likely to have had breast cancer (OR 8.453, 95% CI 1.638-43.606), among other diseases such as melasma, hypertension, and hyperthyroidism. Regarding male patients with rosacea, there was no significant increase in breast cancer incidence. A meta-analysis of observational studies on the association between rosacea and breast cancer was ultimately not conducted because of heterogeneity between the studies; however, it would seem that most of the current data available point to a positive correlation [12].

Owing to the directionality of the studied relationship, we were unable to include in the meta-analysis a case-control study by Long et al [38] that looked at rosacea incidence in various cancers in China. However, they found that patients with breast cancer had a significantly higher incidence of rosacea compared with individuals without rosacea (OR 5, 95% CI 4.02-6.2) [38]. Although this is the only study that was found of the inverse relationship, the potential for a bidirectional association lends further credence to the hypothesis that these 2 conditions are related.

### Rosacea and Melanoma

A total of 8% (3/39) of the studies analyzed the association between rosacea and malignant melanoma. Egeberg et al [6] found no increased occurrence of melanoma in patients with rosacea (HR 1.10, 95% CI 0.95-1.27). Similarly, Li et al [7] also found no significant increase in the development of malignant melanoma in patients with rosacea (RR 0.96, 95% CI 0.57-1.62). Finally, the findings of Cho et al [11] noted that patients with rosacea had no association with the development of melanoma compared with the reference population (HR 1.69, 95% CI 0.25-11.37).

Although excluded from the meta-analysis because of not being in full text, an abstract by Erickson et al [13] investigated sex-specific differences in patients with rosacea and their likelihood of developing certain cancers. The study included 11,466 patients with rosacea, 8676 of whom were female, and 175,363 patients who did not have rosacea, 929 of whom were female. Interestingly, this abstract found that rosacea was inversely associated with the development of melanoma in female patients (RR 0.63, 95% CI 0.47-0.85). No significant relationship was found in male patients with rosacea [13]. Overall, despite the small number of studies available, there was no evidence of an association between the 2 conditions.

### Rosacea and Hepatic Cancer

A total of 5% (2/39) of studies were found that examined the association between rosacea and hepatic cancer. Egeberg et al [6] noted that patients with rosacea have an increased risk of developing hepatic cancer (HR 1.42, 95% CI 1.06-1.90). The findings of Cho et al [11] noted that patients with rosacea had no association with the development of hepatic cancer compared with the reference population (HR 1.32, 95% CI 0.89-1.95).

### Rosacea and Glioma

In total, 3% (1/39) of studies were found that examined the association between rosacea and glioma. Egeberg et al [3] conducted a cohort study on Danish citizens aged >18 years on January 1, 1997, and followed them through to December 31, 2011. Patients with rosacea or glioma at baseline were excluded to allow for the study of the temporal relationship between the conditions. The reference population for the study was 5,416,138 individuals with a mean age of 40.8 (SD 19.7) years, and the rosacea diagnosis group contained 68,372 patients with a mean age of 42.2 (SD 16.5) years. The reference population consisted of 2,684,509 male patients and 2,732,029 female patients. Although the correlation was weak, the study found a significantly increased risk of glioma in patients with rosacea (incidence rate ratio 1.36, 95% CI 1.18-1.58). Interestingly, it was found that the rosacea-associated increased risk of glioma was greater in males than in females [3].

In addition, the excluded case-control study by Long et al [38] found that patients with glioma were more likely to have had rosacea (OR 2.16, 95% CI 1.12-4.17), pointing to a potential bidirectional relationship between the 2 conditions.

### Rosacea and Thyroid Cancer

A total of 5% (2/39) of the studies analyzed the association between rosacea and thyroid cancer, reporting conflicting results. Egeberg et al [6] found no significant increase in the development of thyroid cancer in patients with rosacea (HR 1.06, 95% CI 0.68-1.65), whereas Li et al [7] found an increase in the development of thyroid cancer in patients with rosacea (RR 1.59, 95% CI 1.07-2.36).

## Discussion

### Summary of Principal Findings

Our review found that, based on the available literature, there is a positive association between rosacea and glioma, NMSC, and breast cancer. Rosacea was not found in any study to be significantly associated with melanoma. Regarding BCC, SCC, and thyroid and hepatic cancers, a clear conclusion could not be drawn because of conflicting results across 2 studies.

Our review found conflicting evidence regarding the association between BCC and rosacea. Although Li et al [7] found a positive association between BCC and rosacea in a cohort study of female patients, Lin et al [10] found no association between BCC of the body and rosacea in female patients and an inverse association between BCC of the face and rosacea in female patients. More studies will be needed to determine whether an association truly exists. Interestingly, across our reviewed cohort studies, no positive associations were found between rosacea and facial BCC or facial SCC. This may be due to patients with rosacea adopting better sun-protective measures such as sunscreen and broad hats to avoid flaring their rosacea, simultaneously reducing their risk of facial NMSC. It should be noted that there are currently no comparative studies analyzing the association between BCC and rosacea in male patients. Our review also found 14 case reports and case series discussing the presence of BCC in male patients with rhinophyma. It is uncertain whether the increased prevalence of BCC in this population is due to the male sex, the increased severity of rosacea in patients with rhinophyma, or both. Although rosacea is more commonly seen in females, rhinophyma, a subtype of rosacea, is found to be more common in White males aged >50 years. Although the reason for this is unknown, it is important to note that, according to the current literature, males with rhinophyma are 3% to 10% more likely to develop some form of skin cancer at the site of the nodular lesion [39]. Characteristics to be watchful for in patients with long-standing rhinophyma include sudden changes in ulceration of the lesion and rapid growth associated with malodorous drainage [15].

Our review found a positive association between rosacea and NMSC. It should be noted that actinic keratosis was also found to be associated with rosacea in one cohort study [11]. UV exposure is thought to be a common pathogenic factor in the development of skin cancer, actinic keratosis, and rosacea. It is thought that patients with rosacea have an altered skin barrier and are more likely to have had higher UV exposure at early ages, which may predispose them to skin cancers such as SCC and BCC [3,7]. UV radiation is a known risk factor for skin cancers and also plays an important chronic role in rosacea development through the generation of reactive oxygen species and cathelicidin expression, in addition to being a known acute trigger for rosacea outbreaks [40]. It should be noted that, although NMSC had a positive association with rosacea, SCC showed conflicting results. Although Lin et al [10] found an overall positive association between SCC and rosacea, Li et al [7] found no such association. The conflicting findings between studies on NMSC versus BCC and SCC when it comes to rosacea may be because all current comparative studies regarding BCC, SCC, and rosacea have exclusively used female patients. In comparison, Cho et al [11] found an increased risk of NMSC in patients with rosacea in a cohort that included male patients with SCC and BCC. It is possible that there are gender-specific differences when it comes to rosacea and NMSC, perhaps because of different sun-avoidant practices when having rosacea. In addition, although many studies on rosacea, NMSC, BCC, and SCC controlled for age, smoking status, BMI, and alcohol intake among other potential confounders, race and socioeconomic status were brought up as additional possible confounders that were not adjusted for. More studies are needed to analyze the impact of sex, racial, and socioeconomic differences when it comes to rosacea and NMSCs.

It should be noted that, regarding melanoma, all comparative studies (3/3, 100%) analyzed in our review found no association between melanoma and rosacea. Wu et al [41] proposed that the risk of SCC and BCC is positively associated with cumulative UV exposure, whereas melanoma tends to be more associated with intermittent UV exposure. Therefore, it is possible that cumulative UV radiation may be an important confounding factor in the development of both skin cancers and rosacea, accounting for the seemingly positive association that we observed between rosacea and NMSC or SCC but not melanoma.

Other than one study indicating no association, the relationship between rosacea and breast cancer seemed to be positive. Given the prevalence of both rosacea and breast cancer in females, there may be a component of hormonal changes and estrogen mediating some common inflammatory and immune-related causes [38]. However, there was conflicting evidence regarding the association between hepatic cancer and rosacea. Egeberg et al [6] found a positive association between hepatic cancer and rosacea; however, this result was potentially confounded by the fact that the rosacea group also reported greater alcohol consumption than the controls. Regarding glioma, underlying the increased odds of glioma development in patients with rosacea may be common inflammatory pathways dependent on matrix metalloproteinases and the activation of interleukin-17. In particular, matrix metalloproteinase-9 plays an important role in both rosacea pathogenesis and regulation of cell invasion in malignant glioma [3]. One study found increased expression of matrix metalloproteinase-9 in tumor tissue specimens from 76% of patients with glioblastoma, the most common and aggressive malignant form of glioma [42]. Interleukin-17 upregulation is also recognized as a hallmark of rosacea and may play a role in immune suppression in glioma [43,44].

Studies examining the association between rosacea and thyroid cancer reported conflicting results. Accumulating evidence demonstrates that chronic inflammation plays a pivotal role in the pathogenesis of thyroid cancer [45,46]. Therefore, inflammation may be a potential link between rosacea and thyroid cancer. However, more studies need to be conducted to clarify this relationship.

### Limitations

Our study was not able to conduct a meta-analysis because of the statistical heterogeneity between the studies. A meta-analysis would have further clarified the association between rosacea and the various cancers. In addition, there were a small number of studies on most of the cancers included in our review. Other limitations include a lack of studies examining the association between rosacea and cancer in skins of color. Most of the research described in this review was conducted on White, middle-aged, and female populations; further studies are needed regarding rosacea and cancer incidence in skins of color and male populations. In addition, the field of literature regarding BCC and rhinophyma or ocular rosacea is shallow and only comprises case reports/case series. These case series or case reports were included; however, they have an inherent bias because of the absence of a comparative group.

### Conclusions

Our review of the current literature found that rosacea is significantly associated with NMSC, glioma, and breast cancer. An association between rosacea and thyroid cancer as well as between rosacea and hepatic cancer was also reported, but more studies are needed because of the limited amount of data. Rosacea does not appear to be associated with melanoma. Further studies should be conducted to determine whether there is an association between thyroid cancer and rosacea.

## Appendix

Multimedia Appendix 1 Search strategies.

## Abbreviations

BCC: basal cell carcinoma
HR: hazard ratio
NMSC: nonmelanoma skin cancer
OR: odds ratio
PRISMA: Preferred Reporting Items for Systematic Reviews and Meta-Analyses
RR: risk ratio
SCC: squamous cell carcinoma
