# Supplementary material for: Rosacea and Its Association With Malignancy: Systematic Review
Source: JMIR Dermatol. 2023 Nov 8;6:e47821. doi: 10.2196/47821 (PMC10666011; doi:10.2196/47821)
Supplement: Multimedia Appendix 1 [file derma_v6i1e47821_app1.docx]

| **Supplemental Table I.** Search strategies. |
| --- |
| **OVID MEDLINE : Breast** inception-March 3, 2021. 4 results.  Basal Cell March 3, 2021. 2 results  Squamous Cell March 3, 2021. 3 results  Markel March 3, 2021.1 result  NMSC March 3, 2021.1 result  Melanoma March 3, 2021.10 results  Glioma March 3, 2021.1 result  Hepatic March 3, 2021.3 results  Thyroid March 3, 2021.2 results   1. exp Rosacea/ 2. rosacea.ti,ab. 3. Rhinophyma.ti,ab. 4. pyoderma faciale.ti,ab. 5. 1 or 2 or 3 or 4 6. exp breast/ 7. exp breast disease/ 8. breast cancer/ or breast adenocarcinoma/ or breast carcinoma 9. exp breast tumor/ 10. exp breast cancer/ 11. exp breast carcinoma/ 12. (breast$ adj5 (neoplas$ or cancer$ or carcin$ or tumo$ or metasta$ or malig$)).ti,ab. 13. 6 or 7 or 8 or 9 or 10 or 11 or 12 14. 5 and 13 15. exp Carcinoma, Basal Cell 16. exp Neoplasms, Basal Cell/ 17. Basal Cell Nevus Syndrome/ 18. Bcc.ti,ab. 19. (basal cell$ and (cancer$ or epithelioma$ or carcinoma$ or naev$ or nev$ or tumor$ or tumour$ or neoplasm$)).mp. 20. 16 and 17 and 18 and 19 and 20 and 21 21. 5 and 22 22. carcinoma, squamous cell/ or neoplasms, squamous cell/ 23. exp squamous cell carcinoma/ 24. exp epidermoid carcinoma/ 25. Scc.ti,ab. 26. 24 or 25 or 26 or 27 or 28 27. 5 and 29 28. carcinoma, merkel cell/ or neoplasms, merkel cell/ 29. exp merkel cell carcinoma/ 30. mcc.ti,ab. 31. 31 or 32 or 33 32. 5 and 34 33. Nmsc.ti,ab. 34. 5 and 33 35. exp Melanoma/ 36. melanoma$.ti,ab. 37. malignant melanoma$.ti,ab. 38. 41 or 42 or 43 39. 5 and 44 40. exp Glioma/ 41. exp Astrocytoma/ 42. exp Glioblastoma/ 43. exp Diffuse Intrinsic Pontine Glioma/ 44. exp Ependymoma/ 45. exp Glioma, Subependymal/ 46. subependymoma.mp. 47. exp Ganglioglioma/ 48. exp Gliosarcoma/ 49. exp Medulloblastoma/ 50. exp Oligodendroglioma/ 51. exp Optic Nerve Glioma/ 52. glial tumor.mp. 53. glial cancer.mp. 54. glial carcinoma.mp. 55. glial neoplasm.mp. 56. 46 or 47 or 48 or 49 or 50 or 51 or 52 or 53 or 54 or 55 or 56 or 57 or 58 or 59 or 60 or 61 57. 5 and 62 58. exp Liver Neoplasms/ 59. exp Adenoma, Liver Cell/ 60. exp Carcinoma, Hepatocellular/ 61. exp Hepatoblastoma/ 62. hepatic adenoma.mp. 63. hepatic cancer.mp. 64. hepatic neoplasm.mp. 65. hepatocellular cancer.mp. 66. hepatocellular neoplasm.mp. 67. liver cancer.mp. 68. liver carcinoma.mp. 69. 64 or 65 or 66 or 67 or 68 or 69 or 70 or 71 or 72 or 73 or 74 70. 5 and 75 71. exp Thyroid Neoplasms/ 72. exp Thyroid Cancer, Papillary/ 73. exp Thyroid Nodule/ 74. Hurthle cell carcinoma.mp. 75. Hurthle cell cancer.mp. 76. Hurthle cell neoplasm.mp. 77. exp Carcinoma, Medullary/ 78. Medullary thyroid cancer.mp. 79. Medullary thyroid neoplasm.mp. 80. exp Thyroid Carcinoma, Anaplastic/ 81. Anaplastic thyroid cancer.mp. 82. Anaplastic thyroid neoplasm.mp. 83. exp Thyroid Cancer, Papillary/ 84. Papillary thyroid neoplasm.mp. 85. exp Adenocarcinoma, Follicular/ 86. Follicular thyroid cancer.mp. 87. Follicular thyroid neoplasm.mp. 88. 77 or 78 or 79 or 80 or 81 or 82 or 83 or 84 or 85 or 86 or 87 or 88 or 89 or 90 or 91 or 92 or 93 89. 5 and 94 |
| **EMBASE -** Breast March 3, 2021. 63 results  Basal Cell March 3, 2021. 685 results  Squamous Cell March 3, 2021. 121 results  Merkel March 3, 2021. 682 results  NMSC March 3, 2021. 4 results  Malignant March 3, 2021. 215 results  Glioma March 3, 202. 15 results  Hepatic March 3, 2021. 114 results  Thyroid March 3, 2021. 51 results   1. ‘rosacea’ 2. ‘rhinophyma’ 3. ‘pyoderma faciale’ 4. 1 or 2 or 3 5. 'breast neoplasm' 6. 'breast cancer'/exp OR 'breast cancer' 7. 'breast carcinoma'/exp OR 'breast carcinoma' 8. 'breast neoplasia’ 9. 'breast tumour' 10. 'breast tumor'/exp OR 'breast tumor' 11. 5 or 6 or 7 or 8 or 0 or 10 12. 4 and 11 13. ‘Basal Cell Carcinoma’ 14. ‘basal cell’ and ‘cancer’ or ‘carcinoma’ or ‘naev’ or ‘nev’ or ‘tumor’ or ‘tumour’ or neoplasms’ 15. Bcc.ti,ab. 16. basalioma$.mp. 17. 13 or 14 or 15 or 16 18. 4 and 17 19. ‘squamous cell carcinoma’ 20. ‘squamous cell carcinoma’ 21. ‘epidermoid carcinoma’ 22. ‘squamous cell epithelioma’ 23. Scc.ti,ab. 24. 19 or 20 or 21 or 22 or 23 25. 4 and 24 26. ‘Merkel Cell Carcinoma’ 27. ‘merkel’ and ‘cancer’ or ‘carcinoma’ or ‘naev’ or ‘nev’ or ‘tumor’ or ‘tumour’ or ‘neoplasms’ 28. Mcc.ti,ab. 29. 26 or 27 or 28 30. 4 and 29 31. ‘nonmelanona skin cancer’ 32. ‘Nmsc’ 33. 31 or 32 34. 4 and 33 35. ‘Melanoma’ 36. ‘malignant melanoma’ 37. 36 or 37 38. 4 and 38 39. 'glioma'/exp OR glioma 40. 'astrocytoma'/exp OR astrocytoma 41. ‘glioblastoma’/exp OR glioblastoma 42. 'diffuse intrinsic pontine glioma'/exp OR 'diffuse intrinsic pontine glioma' OR (diffuse AND intrinsic AND pontine AND ('glioma'/exp OR glioma)) 43. 'ependymoma'/exp OR ependymoma 44. 'subependymal glioma'/exp OR 'subependymal glioma' OR (subependymal AND ('glioma'/exp OR glioma)) 45. 'subependymoma'/exp OR subependymoma 46. 'ganglioglioma'/exp OR ganglioglioma 47. 'gliosarcoma'/exp OR gliosarcoma 48. 'medulloblastoma'/exp OR medulloblastoma 49. 'oligodendroglioma'/exp OR oligodendroglioma 50. 'optic nerve glioma'/exp OR 'optic nerve glioma' OR (optic AND ('nerve'/exp OR nerve) AND ('glioma'/exp OR glioma)) 51. 'glial tumor'/exp OR 'glial tumor' OR (glial AND ('tumor'/exp OR tumor)) 52. 'glial cancer' OR (glial AND ('cancer'/exp OR cancer)) 53. 'glial carcinoma' OR (glial AND ('carcinoma'/exp OR carcinoma)) 54. 'glial neoplasm' OR (glial AND ('neoplasm'/exp OR neoplasm)) 55. 40 or 41 or 42 or 43 or 44 or 45 or 46 or 47 or 48 or 49 or 50 or 51 or 52 or 53 or 54 or 55 56. 4 and 56 57. 'liver neoplasms'/exp OR 'liver neoplasms' OR (('liver'/exp OR liver) AND ('neoplasms'/exp OR neoplasms)) 58. 'liver cell adenoma'/exp OR 'liver cell adenoma' OR (('liver'/exp OR liver) AND ('cell'/exp OR cell) AND ('adenoma'/exp OR adenoma)) 59. 'hepatocellular carcinoma'/exp OR 'hepatocellular carcinoma' OR (hepatocellular AND ('carcinoma'/exp OR carcinoma)) 60. 'hepatoblastoma'/exp OR hepatoblastoma 61. 'hepatic adenoma'/exp OR 'hepatic adenoma' OR (hepatic AND ('adenoma'/exp OR adenoma)) 62. 'hepatic cancer'/exp OR 'hepatic cancer' OR (hepatic AND ('cancer'/exp OR cancer)) 63. 'hepatic neoplasm' OR (hepatic AND ('neoplasm'/exp OR neoplasm)) 64. 'hepatocellular cancer' OR (hepatocellular AND ('cancer'/exp OR cancer)) 65. 'hepatocellular neoplasm' OR (hepatocellular AND ('neoplasm'/exp OR neoplasm)) 66. 'liver cancer'/exp OR 'liver cancer' OR (('liver'/exp OR liver) AND ('cancer'/exp OR cancer)) 67. 'liver carcinoma'/exp OR 'liver carcinoma' OR (('liver'/exp OR liver) AND ('carcinoma'/exp OR carcinoma)) 68. 58 or 59 or 60 or 61 or 62 or 63 or 64 or 65 or 66 or 67 or 68 69. 4 and 69 70. 'thyroid neoplasms'/exp OR 'thyroid neoplasms' OR (('thyroid'/exp OR thyroid) AND ('neoplasms'/exp OR neoplasms)) 71. 'thyroid cancer, papillary'/exp OR 'thyroid cancer, papillary' OR (('thyroid'/exp OR thyroid) AND cancer, AND papillary) 72. 'thyroid nodule'/exp OR 'thyroid nodule' OR (('thyroid'/exp OR thyroid) AND ('nodule'/exp OR nodule)) 73. 'hurtle cell thyroid cancer' OR (hurtle AND ('cell'/exp OR cell) AND ('thyroid'/exp OR thyroid) AND ('cancer'/exp OR cancer)) 74. 'hurthle cell carcinoma'/exp OR 'hurthle cell carcinoma' OR (hurthle AND ('cell'/exp OR cell) AND ('carcinoma'/exp OR carcinoma)) 75. 'hurthle cell neoplasm' OR (hurthle AND ('cell'/exp OR cell) AND ('neoplasm'/exp OR neoplasm)) 76. 'medullary thyroid cancer'/exp OR 'medullary thyroid cancer' OR (medullary AND ('thyroid'/exp OR thyroid) AND ('cancer'/exp OR cancer)) 77. 'medullary thyroid carcinoma'/exp OR 'medullary thyroid carcinoma' OR (medullary AND ('thyroid'/exp OR thyroid) AND ('carcinoma'/exp OR carcinoma)) 78. 'medullary thyroid neoplasm' OR (medullary AND ('thyroid'/exp OR thyroid) AND ('neoplasm'/exp OR neoplasm)) 79. 'anaplastic thyroid carcinoma'/exp OR 'anaplastic thyroid carcinoma' 80. 'anaplastic thyroid cancer'/exp OR 'anaplastic thyroid cancer' OR (anaplastic AND ('thyroid'/exp OR thyroid) AND ('cancer'/exp OR cancer)) 81. 'anaplastic thyroid neoplasm' OR (anaplastic AND ('thyroid'/exp OR thyroid) AND ('neoplasm'/exp OR neoplasm)) 82. 'papillary thyroid carcinoma'/exp OR 'papillary thyroid carcinoma' OR (papillary AND ('thyroid'/exp OR thyroid) AND ('carcinoma'/exp OR carcinoma)) 83. 'papillary thyroid neoplasm' OR (papillary AND ('thyroid'/exp OR thyroid) AND ('neoplasm'/exp OR neoplasm)) 84. 'follicular adenocarcinoma'/exp OR 'follicular adenocarcinoma' OR (follicular AND ('adenocarcinoma'/exp OR adenocarcinoma)) 85. 'follicular thyroid cancer'/exp OR 'follicular thyroid cancer' OR (follicular AND ('thyroid'/exp OR thyroid) AND ('cancer'/exp OR cancer)) 86. 'follicular thyroid neoplasm' OR (follicular AND ('thyroid'/exp OR thyroid) AND ('neoplasm'/exp OR neoplasm)) 87. 71 or 72 or 73 or 74 or 75 or 76 or 77 or 78 or 79 or 80 or 81 or 82 or 83 or 84 or 85 or 86 or 87 88. 4 and 88 |
| **COCHRANE CENTRAL:** March 4, 2021.  Breast: 1 results  Basal Cell: 5 results  Squamous Cell: 4 results  Merkel Cell: 0 results  NMSC: 3 results  Malignant Melanoma: 4 results  Glioma: 0 results  Thyroid: 1 result  Hepatic 1 results   1. MeSH descriptor: [Rosacea] explode all trees 2. rosacea:ti,ab 3. rhinophyma:ti,ab 4. "pyoderma faciale":ti,ab 5. #1 or #2 or #3 or #4 6. MeSH descriptor: [Breast Neoplasms] explode all trees 7. (metastatic or advance) and (breast cancer or breast neoplasm or breast carcinoma or breast tumour or breast tumor) 8. breast near cancer* 9. breast near neoplasm* 10. breast near carcinoma* 11. breast near tumour* 12. 6 or 7 or 8 or 9 or 10 or 11 13. 5 and 12 14. MeSH descriptor: [Carcinoma, Basal Cell] explode all trees 15. MeSH descriptor: [Neoplasms, Basal Cell] explode all trees 16. MeSH descriptor: [Basal Cell Nevus Syndrome] explode all trees 17. (basal cell* next (cancer* or epithelioma* or carcinoma* or naev* or nev* or tumor* or tumour* or neoplasm*)):ti,ab,kw 18. Bcc:ti,ab,kw 19. #14 or #15 or #16 or #17 or #18 20. #5 and #19 21. MeSH descriptor: [Carcinoma, Squamous Cell] explode all trees 22. MeSH descriptor: [Neoplasms, Squamous Cell] explode all trees 23. ("squamous cell" next (carcinoma* or epithelioma*)):ti,ab 24. epidermoid next carcinoma*:ti,ab 25. scc:ti,ab,kw 26. #21 or #22 or #23 or #24 or #25 27. #5 and #26 28. MeSH descriptor: [Merkel Cell carcinoma] explode all trees 29. MCC:ti,ab,kw 30. #28 or #29 31. #5 and #30 32. MeSH descriptor: [Nonmelanoma skin cancer] explode all trees 33. Nmsc:ti,ab,kw 34. #32 or #33 35. #5 and #34 36. MeSH descriptor: [Melanoma] explode all trees 37. melanoma*:ti,ab,kw 38. malignant melanoma*:ti,ab,kw 39. #36 or #37 or #38 40. #5 and #39 41. MeSH descriptor: [Glioma] explode all trees 42. MeSH descriptor: [Astrocytoma] explode all trees 43. MeSH descriptor: [Glioblastoma] explode all trees 44. MeSH descriptor: [Diffuse Intrinsic Pontine Glioma] explode all trees 45. MeSH descriptor: [Ependymoma] explode all trees 46. MeSH descriptor: [Glioma, Subependymal] explode all trees 47. Subependymoma 48. MeSH descriptor: [Ganglioglioma] explode all trees 49. MeSH descriptor: [Gliosarcoma] explode all trees 50. MeSH descriptor: [Medulloblastoma] explode all trees 51. MeSH descriptor: [Oligodendroglioma] explode all trees 52. MeSH descriptor: [Optic Nerve Glioma] explode all trees 53. Glial tumor*:ti,ab,kw 54. Glial cancer*:ti,ab,kw 55. Glial carcinoma*:ti,ab,kw 56. Glial neoplasm*:ti,ab,kw 57. #41 or #42 or #43 or #44 or #45 or #46 or #47 or #48 or #49 or #50 or #51 or #52 or #53 or #54 or #55 or #56 58. #5 and #57 59. MeSH descriptor: [Liver Neoplasms] explode all trees 60. MeSH descriptor: [Adenoma, Liver Cell] explode all trees 61. MeSH descriptor: [Carcinoma, Hepatocellular] explode all trees 62. MeSH descriptor: [Hepatoblastoma] explode all trees 63. Hepatic adenoma*:ti,ab,kw 64. Hepatic cancer*:ti,ab,kw 65. Hepatic neoplasm*:ti,ab,kw 66. Hepatocellular cancer*:ti,ab,kw 67. Hepatocellular neoplasm*:ti,ab,kw 68. Liver cancer*:ti,ab,kw 69. Liver carcinoma*:ti,ab,kw 70. #59 or #60 or #61 or #62 or #63 or #64 or #65 or #66 or #67 or #68 or #69 71. #5 and #70 72. MeSH descriptor: [Thyroid Neoplasms] explode all trees 73. MeSH descriptor: [Thyroid Cancer, Papillary] explode all trees 74. MeSH descriptor: [Thyroid Nodule] explode all trees 75. Hurthle Cell thyroid cancer*:ti,ab,kw 76. Hurthle cell carcinoma*:ti,ab,kw 77. Hurthle cell neoplasm*:ti,ab,kw 78. Medullary thyroid cancer*:ti,ab,kw 79. Medullary thyroid carcinoma*:ti,ab,kw 80. Medullary thyroid neoplasm*:ti,ab,kw 81. MeSH descriptor: [Thyroid Carcinoma, Anaplastic] explode all trees 82. Anaplastic thyroid cancer*:ti,ab,kw 83. Anaplastic thyroid neoplasm*:ti,ab,kw 84. Papillary thyroid carcinoma*:ti,ab,kw 85. Papillary thyroid neoplasm*:ti,ab,kw 86. MeSH descriptor: [Adenocarcinoma, Follicular] explode all trees 87. Follicular thyroid cancer*:ti,ab,kw 88. Follicular thyroid neoplasm*:ti,ab,kw 89. #72 or #73 or #74 or #75 or #76 or #77 or #78 or #79 or #80 or #81 or #82 or #83 or #84 or #85 or #86 or #87 or #88 90. #5 and # 89 |
| **PUBMED:**  Breast cancer: inception - 2/21/2021.131 results  Basal 2/21/2021: 443  SSC: 40 results  Markel: 1 results  NMSC: 7 results  Melanoma: 20 results  Glioma: 4 results  Hepatic: 8 results  Thyroid: 3 results   1. "Rosacea"[Mesh] 2. "Rhinophyma"[Mesh] 3. "pyoderma faciale"[Mesh] 4. 1 or 2 or 3 5. "Melanoma"[Mesh] 6. "Breast Neoplasms"[Mesh] 7. "Breast Diseases"[Mesh] 8. Breast cancer 9. Breast neoplasia 10. Breast adenocarcinoma 11. Breast carcinoma 12. Breast tumor 13. Breast tumour 14. Breast metastasis 15. Breast malignancy 16. metastatic or advance 17. breast cancer or breast neoplasm or breast carcinoma or breast tumour or breast tumor 18. 5 or 6 or 7 or 8 or 9 or 10 or 11 or 12 or 13 or 14 or 15 or 16 or 17 19. 4 and 18 20. "Carcinoma, Basal Cell"[Mesh] 21. "Neoplasms, Basal Cell"[Mesh] 22. BCC 23. basal cell or skin or epidermal or cutaneous 24. cancer or carcinoma or mass or masses or tumour or tumor or neoplasm or adenoma or epithelioma or lesion or malignancy or nodule 25. 23 and 24 26. 20 or 21 or 22 or 25 27. 4 and 26 28. "Carcinoma, Squamous Cell"[Mesh] 29. "Neoplasms, Squamous Cell"[Mesh] 30. Squamous cell 31. cancer or carcinoma or mass or masses or tumour or tumor or neoplasm or adenoma or epithelioma or lesion or malignancy or nodule 32. 30 and 31 33. SCC 34. 28 or 29 or 32 or 33 35. 4 and 34 36. "Carcinoma, Merkel Cell"[Mesh] 37. MCC 38. Merkel Cell 39. cancer or carcinoma or mass or masses or tumour or tumor or neoplasm or adenoma or epithelioma or lesion or malignancy or nodule 40. 38 and 39 41. 36 or 37 or 40 42. 4 and 41 43. Nonmelanoma skin cancer 44. NMSC 45. 43 or 44 46. 4 and 45 47. "Melanoma"[Mesh] 48. Malignant melanoma 49. 47 or 48 50. 4 and 49 51. "Glioma"[Mesh] 52. "Astrocytoma"[Mesh] 53. "Glioblastoma"[Mesh] 54. "Diffuse Intrinsic Pontine Glioma"[Mesh] 55. "Ependymoma"[Mesh] 56. "Glioma, Subependymal"[Mesh] 57. Subependymoma 58. "Ganglioglioma"[Mesh] 59. "Gliosarcoma"[Mesh] 60. "Medulloblastoma"[Mesh] 61. "Oligodendroglioma"[Mesh] 62. "Optic Nerve Glioma"[Mesh] 63. Glial tumor 64. Glial cancer 65. Glial carcinoma 66. Glial neoplasm 67. 46 or 47 or 48 or 49 or 50 or 51 or 52 or 53 or 54 or 55 or 56 or 57 or 58 or 59 or 60 or 61 68. 4 and 62 69. "Liver Neoplasms"[Mesh] 70. "Adenoma, Liver Cell"[Mesh] 71. "Carcinoma, Hepatocellular"[Mesh] 72. "Hepatoblastoma"[Mesh] 73. Hepatic adenoma 74. Hepatic cancer 75. Hepatic neoplasm 76. Hepatocellular cancer 77. Hepatocellular neoplasm 78. Liver cancer 79. Liver carcinoma 80. 64 or 65 or 66 or 67 or 68 or 69 or 70 or 71 or 72 or 73 or 74 81. 4 and 75 82. "Thyroid Neoplasms"[Mesh] 83. "Thyroid Cancer, Papillary"[Mesh] 84. "Thyroid Nodule"[Mesh] 85. "Thyroid cancer, Hurthle cell" [Supplementary Concept] 86. Hurthle cell carcinoma 87. Hurthle cell neoplasm 88. "Thyroid cancer, medullary" [Supplementary Concept] 89. Medullary thyroid carcinoma 90. Medullary thyroid neoplasm 91. "Thyroid Carcinoma, Anaplastic"[Mesh] 92. Anaplastic thyroid cancer 93. Anaplastic thyroid neoplasm 94. Papillary thyroid carcinoma 95. Papillary thyroid neoplasm 96. "Adenocarcinoma, Follicular"[Mesh] 97. Follicular thyroid cancer 98. Follicular thyroid neoplasm 99. 77 or 78 or 79 or 80 or 81 or 82 or 83 or 84 or 85 or 86 or 87 or 88 or 89 or 90 or 91 or 92 or 93 100. 4 and 94 |

**Breast**

All results before deduplication: 199

After Endnote deduplication: 192

Total duplicates: 7

After Rayyan Deduplication: 191

Abstracts screened:

Records excluded:

Full text articles assessed for eligibility:

Full text articles excluded with reasons:

Studies included for quantitative synthesis and meta-analysis:

**Basal Cell**

All results before deduplication: 1135

After Endnote deduplication: 1025

Total duplicates: 110

After Rayyan Deduplication: 1007

Abstracts screened:

Records excluded:

Full text articles assessed for eligibility:

Full text articles excluded with reasons:

Studies included for quantitative synthesis and meta-analysis:

**Squamous Cell**

All results before deduplication: 168

After Endnote deduplication: 152

Total duplicates: 16

After Rayyan Deduplication: 147

Abstracts screened:

Records excluded:

Full text articles assessed for eligibility:

Full text articles excluded with reasons:

Studies included for quantitative synthesis and meta-analysis:

**Merkel**

All results before deduplication: 684

After Endnote deduplication: 677

Total duplicates: 7

After Rayyan Deduplication: 672

Abstracts screened:

Records excluded:

Full text articles assessed for eligibility:

Full text articles excluded with reasons:

Studies included for quantitative synthesis and meta-analysis:

**NMSC**

All results before deduplication: 15

After Endnote deduplication: 13

Total duplicates: 2

After Rayyan Deduplication: 13

Abstracts screened:

Records excluded:

Full text articles assessed for eligibility:

Full text articles excluded with reasons:

Studies included for quantitative synthesis and meta-analysis:

**Melanoma**

All results before deduplication: 249

After Endnote deduplication: 229

Total duplicates: 20

After Rayyan Deduplication: 223

Abstracts screened:

Records excluded:

Full text articles assessed for eligibility:

Full text articles excluded with reasons:

Studies included for quantitative synthesis and meta-analysis:

**Glioma**

All results before deduplication: 20

After Endnote deduplication: 15

Total duplicates: 5

After Rayyan Deduplication: 15

Abstracts screened:

Records excluded:

Full text articles assessed for eligibility:

Full text articles excluded with reasons:

Studies included for quantitative synthesis and meta-analysis:

**Hepatic**

All results before deduplication: 126

After Endnote deduplication: 118

Total duplicates: 8

After Rayyan Deduplication: 114

Abstracts screened:

Records excluded:

Full text articles assessed for eligibility:

Full text articles excluded with reasons:

Studies included for quantitative synthesis and meta-analysis:

**Thyroid**

All results before deduplication: 57

After Endnote deduplication: 53

Total duplicates: 4

After Rayyan Deduplication: 53

Abstracts screened:

Records excluded:

Full text articles assessed for eligibility:

Full text articles excluded with reasons:

Studies included for quantitative synthesis and meta-analysis:

2,435 papers
